# 

**Published:** 1850-03

**Authors:** 


### ARTICLE VI.

### TO SUBSCRIBERS.

With the last number of the Journal, we sent the bills of our delinquent subscribers, many of which must have been lost out by Post Masters, as we have not received any return from them. A few, however, went safely, and we most cordially return our thanks to our friends, who have thus promptly responded to the call that necessity forced us to make. Their names will be found on the cover. And if any this number, do not find an acknowledgment of it in the list, who remitted in time for it to have arrived before the issue of they should notify us through the Post Master. But we trust that our friends who have not remitted their dues, will do it at once. Certainly, publishing at a price barely sufficient to pay the printer, and having complied faithfully, with our part of the contract, makes that that is due us our property, and gives us a right to claim it.

☞ Those who pay their dues, before the issue of the next number, can pay for this Volume with Two Dollars; but if delayed until after that time, we shall invariably claim the additional Dollar as the only means of justifying ourselves in continuing the publication. And we assure our friends, we would prefer to receive the Two Dollars now, rather than Three, at a future period.

If any of our subscribers wish to discontinue the work, they will please notify us through the Post Master, or *post-paid*, before the issue of the next number, as we do not discontinue except at the close of the Volume. It is exceedingly unfair to allow us to send the first number of a volume, and then refuse to take it out; for it spoils the number, and unless the Post Master marks it *returned*, it costs us postage.

All letters must hereafter be *post paid*, or they will not be taken out of the office. Those sending unpaid letters therefore, may know why they do not receive attention.

### NOTICE TO READERS AND CORRESPONDENTS.

We have received Original Communications from Drs. R. Willard, I. E. Thayer, C. A. Hathwell, N. Steel, and E. D. Chambers, and an Obituary Notice of Dr. Pratt, of Ottawa, by Dr. Howland, which was designed for insertion in this number, but was unavoidably crowded out. It shall appear in our next. Also, an anonymous communication, which we decline publishing, until the author's name is furnished us.

Our friend Dr. Buckner, has shown us a pamphlet by Dr. Latta, on the report of the Homœopathic Physicians, of Cincinnati, and that of their packed Committee appointed to examine the charges made against them, in the Methodist Expositor, which we published in a former number of this Journal. The pamphlet exposes in a manner not to be misunderstood or gainsaid, the miserable trickery to which these quacks resort, for the purpose of bolstering up their system of empiricism. We will, in the next number, lay before our readers a synopsis of this controversy.

Our thanks are due Messrs. Tilden & Co., of New York, for a package of very handsomely put up Medicinal Extracts. If their quality should equal their appearance, they may certainly be pronounced superior to any thing of the kind we have yet seen. Should they stand the test to which they are now being subjected, we will most cheerfully recommend them to the patronage of Physicians and Apothecaries. Any improvement in Pharmaceutical Preparations is a service done both to the profession and community.

In publishing the Proceedings of the Ottawa Medical Society, in our last No., the name of the Secretary elect, was printed *D. D. Morrison:* it should have been D. D. Thompson. The error was made by the proof-reader. In the present No. a vexatious mistake occurs. The pages from 525 to 541, are numbered incorrectly. The error was not detected until the entire edition was worked off.

### INDEX.

Abscess in the substance of the Brain, 5[illegible]3
Addresses, Medical, 59
Æsculapean Society, proceedings, 449
American Medical Association, 160 541
Allaire on Convulsions of Children during paroxysms of Intermittent fever, 206
Aneurism, Brainard's case of Popliteal 28
Anæsthesia, 312
Asthma cured by external applications, 391
Bailey's Report on the Drug Law, 349
Ballard on Fevers, 93
Banks' case of Medullary Exostosis, 26
" on Pneumonia in Miasmatic Districts, 392
Barth and Rogers on Auscultation and Percussion, notice of. 339
Beck,s case of Nasal Polypus, 122
Bowman's Chemistry, notice of, 327
Brackett on preparation of Mercurial Ointment. 390
Brainard's case of "Extroduction of the bougie," 21
Brainard's case of Popliteal Aneurism, 28
" on treatment of Nævus. 244
" on Chloroform in Periodical Neuralgia, 286
" on Ulceration of the Vagina, 308
Bull on Maternal management of children, notice of, 292
Bullard on masked Intermittents, 197
Channing on Medical Electricity, notice of. 66
Channing on Etherization in Childbirth, notice of, 289
Chicago Marine Hospital, 175
Cholera, treatment of Malignant, 74
" Treatment of, 75
" Contagion of, 78
" The, 178
" Herrick on, 181
" Ritchie's Review of Herrick on 220
" McArthur on, 230
" Spread, and Communicable Nature of, 245
" Infantum, Ritchey on, 298
" on Animalculæ in the Atmosphere of. 411
" Cryptogamous Origin of, 426
" Davis on, 482
Cod Liver Oil, 78
" " " in Phthisis, 415
Collodion as an application in Erysipelas, 386
Concours, the 458
Congelation as a remedy, 71
Convulsions of children in Intermittent Fever, 206
Convulsions of children treated with Quinine, 352
Coroner's Inquest, 118
Coventry on Cholera, notice of, 124
Croup, Nitrate of Silver in, 108
Davis on Cholera, 482
Day on Old Age, notice of, 291
Demonstrator of Anatomy in Rush Medical College, 457
Diphtherite treated with Ammonia and Oil, 144
Dowler's contributions to Physiology, notice of, 405
Dysentery, Nitrate of Silver in Sub-Acute. 388
Eclampsia Parturientium, Hutchinson on, 469
Editorial Department, 91
Electricity, medical application of, 14
Encephalitis, Hall's case of 212
Enuresis, use of Belladonna in, 149
Ergot in Post-partum Hemorrhage, 76
Ether, claim to discovery of Anæsthetic property of, 56
Evans on Cholera, 245
Exostosis, Banks' case of, 26
Fever, Howland on, 3
" Congestive, remarks on, 24
" of summer and winter; causes and treatment of, 93
" Typhoid, observations on 104
Fœtus, accidental expulsion of, without injury, 77
Foreign bodies, new mode of expelling 91
Fox River Medical Society, 517
Free Medical Schools, 362-365-440
Freer on Collodion in Erysipelas, 386
Goff's case, 118
Guaicum in Dysmenorrhœa, 473
Hair-cutting, influence on health, 149
Hall's case of Encephalitis, 212
Hansford on foreign bodies in the larynx, 91
Hastings' Surgery, notice of, 343
Hathwell on Congestive Fever, 24
Heart-Clot, Meigs on, 133
Heart, Hypertrophy of, with valvular disease, 372
Hemorrhoids treated with Nitric Acid, 435
Hendrick's Letter, 109
Herrick on Cholera, 181
Homœopathy in Cincinnati, 356
Homœopathist, trial for manslaughter 533-[4[illegible]3]
Howland on Fever, 3
Hutchinson on Puerperal Convulsions, 469
Intermittents, Masked, Bullard on, 197
Illinois State Medical Convention, 455-456
Jackson on Electricity, 14
Jarvis on Dislocation of the Shoulder, notice of, 54
Journal, retrospective abstract of, 79
Kirke's & Pagets Physiology, notice of, 326
Latta's Circular, 459
Lead Colic, two cases of, 467

Lee's Clinical Midwifery, notice of 342
Lescher on Pneumonia Miasmatica, 509
Logan on Trembles or Animal poison, 380
Lunatic Hospital Reports, 62
Lung, Ossification of, 238
McArthur on Cholera, 230
" " Medical Legislation, 475
McLean on Hygiene, 462
Malaria, modifying influence in the south and west, 30
Malpractice, suit for, 227
Matteucci's Lectures, review of 34
Matthews on Typhoid Fever, 104
" case of Scarlatina, 306
" on Sub-Acute Dysentery, 388
Mead's case of Ovarian Dropsy, 13
Medical Schools, statistics of 90
Medical Miscellany, 92 180 297-370 461 546
Medicine, Sectional Teaching of, 111
Medical Societies, 364
Meig's Obstetrics, notice of 131
Milk-sickness, Logan on 380
Mitchell on Fevers, review of, 328
Morton's Anatomy, notice of 57
Muriate of Opium, 148
Nævus treated with collodion, 244
Neuralgia, Periodical, cured by chloroform, 286
New Medical Journals, 542
Noad's Chemical Analysis, notice of 344
Nofsinger on Toxicology, 498
Noyes' case of Puerperal Convulsions 19
Nux Vomica in Intestinal Obstructions 362
Obituary, 296-370-461
Our Journal, 545
Ovary, Dropsy of, 13
Ozone, Spengler on, 146
Patent Medicine, 151
Pharmacopœia, Convention for revising 177
Pneumonia of Miasmatic Districts, Banks on, 392
Pneumonia of Miasmatic Districts, Lescher on, 509
Polypus, Nasal, treated by local application of peach leaves, 122
Pomological Convention, notice of proceedings of, 519
Pseudo-Membranous, Laryngitis, 68
Puerperal Convulsions, case of, 19
Quain & Sharpey's Anatomy, notice of, 345
Ritchey on Cholera Infantum, 298
Rock-River Medical Soociety, 242
Rush Medical College, 176
Satterlee on Croup, 108
Scarlatina, Matthew's case of, 306
" treated by Inunction, 536-[436]
Sedgwick on Malaria, 30
Simpsons Anæsthesia in Surgery and Midwifery, notice of, 132
Smith on Parturition, notice of, 292-400
Spencer's Report of Suit for Malpractice 227
" " " cases of Lead cholic 467
Stahl's Sectional Teachings of Medicine 111
Stanley's Treatise on Diseases of the Bones, notice of, 410
State Medical Associations, 294-544
Stone on Dysmenorrhœa, 473
Therapeutic use of Hygienic Measures 462
Thompson's case of Hypertrophy of the Heart, 372
Thompson's Conspectus, notice of, 522
Tooth-Ache, new remedy for, 77
Townsend on Asthma, 391
Toxicology, Nofsinger on, 498
To Subscribers, 547
Transactions of the College of Physicians of Philadelphia, 56
Twins, locked in each other, 70
Ungt. Hydrag., preparation of, 390
Urethra, Brainard's case of Stricture of, 21
Uterine Hemorrhage, compression of Aorta in, 438
Vagina, Ulcerations of, 308
Warren on Chloroform & Chloric Ether, notice of, 289
Warren on Etherization, notice of, 290
West on Diseases of Children, notice of, 402
White on Obstetrical Forceps, notice of, 404
Williams & Johnson on Ossification of the Lung, 238

### LIST OF RECEIPTS FOR JANUARY & FEBRUARY 1850.

ILLINOIS.

| | |
|---|---|
| Drs. W. B. Norton, | $3 00 |
| J. V. Goltra, | 2 00 |
| D. D. Thompson, | 2 00 |
| Capt. R. K. Swift, | 2 00 |
| J. E. Parker, | 4 00 |
| A. B. DeWolf, | 4 00 |
| B. Robinson, | 2 00 |
| W. LeBaron, | 1 00 |
| J. R. Stone, | 2 00 |
| G. S. Barrows, | 5 00 |
| R. F. Henry, | 2 0 |
| P. T. Leeds, | 4 00 |
| B. K. Hart, | 4 00 |
| Geo. S. Wheeler, | 1 00 |
| Geo. Haskell, | 2 00 |
| L. Sawyer, | 4 00 |
| H. Martin, | 2 00 |
| W. C. Snyder, | 2 00 |
| T. Wilkins, | 2 00 |
| F. B. Haller, | 2 00 |
| M. D. Sweetland, | 2 0 |
| Wm. Sweetland, | 2 00 |
| A. Hull, | 2 00 |
| E. Woodruff, | 5 00 |
| C. A. Hathwell, | 2 00 |
| R. S. Lord, | 2 00 |
| Z. H. Madden, | 2 00 |
| W. J. Duckwall, | 2 00 |
| J. A. Holderman, | 2 00 |
| S. Wiley, | 2 00 |
| J. Lesher, | 2 00 |
| P. Schemerhorn, | 4 00 |
| A. Hager, | 2 00 |
| Dan'l Ward, | 4 00 |
| J, Bunce, | 2 00 |
| Rev. R. A. Blanchard, | 1 00 |
| J. E. McGirr, | 2 00 |
| B. S. Robinson, | 2 00 |

INDIANA.

| | |
|---|---|
| [illegible]s. A. L. Pettyjohn, | $2 00 |
| A. Buchanan, | 6 00 |
| W. A. Sanger, | 2 00 |
| M. D. Swiggett, | 1 00 |
| W. B. Smith, | 4 00 |
| John Lewis, | 2 00 |
| J. Hodson, | 2 00 |
| J. P. Russell, | 2 00 |
| J. E. Moler, | 2 00 |
| Drs. Geo. Manners, | $4 00 |
| W. F. Boor, | 2 00 |
| E. D. Chambers, | 2 00 |
| S. B. Kiler, | 2 00 |
| M. W. Gentry, | 4 00 |
| Robt. Stevens, | 4 00 |
| J. W. McKinney, | 2 00 |
| — Grubbs, | 2 00 |
| J. O. Wade, | 4 00 |
| R. Curran, | 4 0 |
| Alvin Carley, | 2 00 |
| W. Lockhart, | 2 00 |
| R. Willard, | 2 00 |
| Newkirk, | 4 0 |

MICHIGAN.

| | |
|---|---|
| Drs. Jno. McLean, | $2 00 |
| Jno. Acres, | 2 00 |
| A. R. Calkins, | 2 00 |
| Stillman & Alger, | 2 00 |
| A. P. Alger, | 1 0 |
| Matthew Gill, | 2 00 |
| W. B. Lincoln, | 2 00 |
| C. B. Goodrich, | 2 00 |
| J. S. Skinner, | 1 0 |
| E. D. Burr, | 2 00 |
| J, Goncher, | 3 00 |

IOWA.

| | |
|---|---|
| Drs. Jno. F. Henry, | $2 00 |
| N. Steel, | 2 00 |
| W. L, Orr, | 2 00 |
| D. Dickerson, | 2 00 |

WISCONSIN.

| | |
|---|---|
| Drs. O. T. Maxson, | $1 00 |
| C. C. Warner, | 2 00 |
| D. Sibley, | 2 00 |
| G. M. House, | 2 00 |
| J. M. Todd, | 2 00 |
| J. C. Mills, | 5 00 |
| I. R. Rood, | 1 00 |
| Hays McKinley, | 2 00 |
| R. Dunlap, | 2 00 |

OHIO.

| | |
|---|---|
| Drs. Elias Fisher, | $6 00 |
| S. Stough, | 2 00 |
| L. Gishwiller, | 2 00 |

DELAWARE.

| | |
|---|---|
| Dr. Jas. Couper, | $5 00 |

NEW YORK.

| | |
|---|---|
| Dr. R. Scott, | $5 00 |

### VACCINE VIRUS

Can be obtained by sending One Dollar, post-paid, to the address of the Editors of this Journal. The Virus will be enclosed in a letter, and sent by the return mail.

### PROSPECTUS OF THE THIRD VOLUME OF

### THE NORTH-WESTERN MEDICAL AND SURGICAL JOURNAL.

EDITED BY

JOHN EVANS, M.D.,

PROFESSOR OF OBSTETRICS AND DISEASES OF WOMEN AND CHILDREN IN RUSH MEDICAL COLLEGE, AND

EDWIN G. MEEK, M.D.

This work, as heretofore, will be issued at Chicago and Indianapolis, on the first of every other month, beginning with May, in numbers, each containing from 88 to 96 pages, making at the end of the year, a volume of about 550 pages, octavo, with title page and index complete.

In addition to the usual large amount of original matter, the fourth coming volume will contain a

HISTORY OF THE PROFESSION IN THE UNITED STATES,
FROM THE FIRST SETTLEENT OF THE COLONIES, TO THE PRESENT TIME, by Prof. N. S. Davis, M.D., which of itself will be richly worth the subscription price. No physician who properly regards his profession should fail to secure a copy.

As the list of contributors to the work is annually increasing, it may be expected to furnish as good a variety of interesting original matter as any work of the kind in the country. The diseases of the west will claim especial attention.

Contributions to our pages, especially from physicians of the North-west, are earnestly solicited.

TERMS.---Two Dollars per annum, *in advance*, which may be paid to any of our authorized agents, or sent to us by mail, post paid, at our risk, directed to the "North-Western Medical and Surgical Journal, Chicago, Illinois." If payment be delayed until the close of the volume, three dollars will be invariably charged. No subscription received for less than an entire volume.

A few copies of the FIRST and SECOND VOLUMES remain unsold, and will be sent by mail for **ONE DOLLAR PER. VOLUME;** (the cash to accompany a post-paid order,) or the work will be furnished, half-bound, in sheep, for $1 25 per volume.

☞ A list of receipts will be published in each number.

S. A. Pease, Traveling Agent for Wisconsin.

